# Diversity, distribution and natural *Leishmania* infection of sand flies from communities along the Interoceanic Highway in the Southeastern Peruvian Amazon

**DOI:** 10.1371/journal.pntd.0009000

**Published:** 2021-02-10

**Authors:** Hugo O. Valdivia, Victor O. Zorrilla, Liz. J. Espada, Jocelyn G. Perez, Hugo R. Razuri, Hubert Vera, Roberto Fernandez, Carlos Tong, Bruno M. Ghersi, Gissella M. Vasquez, Roxanne G. Burrus, Andres G. Lescano, Joel M. Montgomery

**Affiliations:** 1 Department of Parasitology, U.S. Naval Medical Research Unit 6, Lima, Peru; 2 Department of Entomology, U.S. Naval Medical Research Unit 6, Lima, Peru; 3 Department of Emerging Infections, U.S. Naval Medical Research Unit 6, Lima, Peru; 4 Dirección Regional de Salud de Madre de Dios, Puerto Maldonado, Peru; 5 Emerge, Emerging Diseases and Climate Change Research Unit, and Clima, Latin American Center of Excellence for Climate Change and Health, Universidad Peruana Cayetano Heredia, Lima, Peru; Instituto Goncalo Moniz-FIOCRUZ, BRAZIL

## Abstract

The Peruvian-Brazilian border is a highly endemic tegumentary leishmaniasis region in South America. The interoceanic highway is a commercial route that connects Peru and Brazil through Madre de Dios and has raised concerns about its impact on previously undisturbed areas. In order to assess leishmaniasis transmission risk along this highway, we conducted a surveillance study of the sand fly populations in this area. Sand flies were collected between 2009 and 2010 along transects at 200 m, 600 m and 1000 m from six study sites located along the highway (Iberia, La Novia, Alto Libertad, El Carmen, Florida Baja, Mazuko and Mavila) and an undisturbed area (Malinowski). Collected specimens were identified based on morphology and non-engorged females of each species were pooled and screened by kinetoplast PCR to detect natural *Leishmania* infections. A total of 9,023 specimens were collected belonging to 54 different *Lutzomyia* species including the first report of *Lu*. *gantieri* in Peru. Four species accounted for 50% of all specimens (*Lutzomyia carrerai carrerai*, *Lu*. *davisi*, *Lu*. *shawi* and *Lu*. *richardwardi*). El Carmen, Alto Libertad, Florida Baja and Malinowski presented higher Shannon diversity indexes (H = 2.36, 2.30, 2.17 and 2.13, respectively) than the most human disturbed sites of Mazuko and La Novia (H = 1.53 and 1.06, respectively). PCR detected 10 positive pools belonging to *Lu*. *carrerai carrerai*, *Lu*. *yuilli yuilli*, *Lu*. *hirsuta hirsuta*, *Lu*. *(Trichophoromyia)* spp., and *Lu*. *(Lutzomyia)* spp. Positive pools from 1,000 m transects had higher infectivity rates than those from 600 m and 200 m transects (9/169 = 5.3% vs 0/79 = 0% and 1/127 = 0.8%, p = 0.018). El Carmen, accounted for eight out of ten positives whereas one positive was collected in Florida Baja and Mazuko each. Our study has shown differences in sand fly diversity, abundance and species composition across and within sites. Multiple clustered *Lutzomyia* pools with natural *Leishmania* infection suggest a complex, diverse and spotty role in leishmaniasis transmission in Madre de Dios, with increased risk farther from the highway.

## Author summary

Leishmaniasis is a neglected disease that is endemic in tropical and subtropical regions affecting up to 2 million people every year. Currently, there is concern about the effects of human-made environmental changes on the transmission of leishmaniasis due to increased exposure to the sand fly vector. In this paper, the authors explored changes in the distribution of sand flies and natural leishmania infection across communities located along the interoceanic highway that connects Peru and Brazil. The study found differences in sand fly species composition and abundance between and within sites as well as a lower sand fly diversity in communities with high human disturbance. Ten pools belonging to *Lutzomyia shawi*, *Lu*. *carrerai carrerai*, *Lu*. *yuilli yuilli*, *Lu*. *hirsuta hirsuta*, *Lu*. *(Helcocyrtomyia)* spp. and *Lu*. *(Lutzomyia)* spp. were found positive for *Leishmania* spp. The majority of these positive pools were collected from a single community and far from the highway. The information provided by this manuscript will serve as a baseline to assess the effects of human activities in the region and guide future surveillance and intervention strategies.

## Introduction

Leishmaniasis is a complex parasitic disease caused by digenetic protozoans belonging to the genus *Leishmania* that is transmitted by the bite of infected phlebotomine sand flies. This disease is endemic in 98 countries with up to 350 million people at risk; causing more than 1.5 million new cases per year with an estimated 12 million people living with the infection [1].

Leishmaniasis is caused by at least 20 different *Leishmania* species and presents a wide spectrum of clinical manifestations that are divided into tegumentary (TL) and visceral leishmaniasis (VL) [2]. In the New World, TL is the most common clinical form of leishmaniasis and causes skin sores that can lead to mutilation and disfigurement. Most of these cases are caused by species of the *Viannia* subgenus, including *L*. *(Viannia) braziliensis*, *L*. *(V*.*) peruviana*, *L*. *(V*.*) guyanensis* and *L*. *(V*.*) panamensis* [3,4].

*Leishmania* transmission relies on the interaction between an infected phlebotomine sand fly of the genus *Phlebotomus* in the Old World or *Lutzomyia* in the New World, with a mammal host [5]. Therefore, the identification of putative *Leishmania* vectors provides key information for epidemiological control and risk assessment in endemic areas.

Peru is one of the ten countries where more than 75% of TL cases worldwide occur, with more than 6,563 cases reported in 2017 [1,6]. The Peruvian Amazon is an endemic leishmaniasis region due to the broad diversity of sand fly vectors, reservoirs and human activities [7]. However, information about the geographic distribution of *Leishmania* vectors, their role in disease transmission and the variables affecting their distribution is still scarce.

The Interoceanic Highway opened in 2005 and is an international route that interconnects Peru and Brazil through Madre de Dios (Peru) and Acre (Brazil) States. Currently, there is major concern about its potential impact on previously undisturbed areas due to human activities and their effects on the diversity, abundance and distribution of leishmaniasis vectors and disease [8,9].

In this study, we conducted sand fly surveillance in communities in Madre de Dios and Puno states along the Interoceanic Highway in Peru and employed molecular tools to identify natural *Leishmania* infections in sand flies collected in this region of the Peruvian-Brazilian Amazon Basin. Our results will serve as a basis for the identification of putative *Leishmania* vectors in this area to better assess the long term impact of human activities conducted along the highway on the transmission of leishmaniasis in the region.

## Methods

### Ethics statement

This study was exempt from NAMRU-6 IRB review as this project did not involve humans as the subject of the study evaluation. Therefore, this study did not meet the definition of research involving human subjects.

Sand fly collections were performed under approval from the General Directorate of Forestry and Wild Fauna from the Ministry of Agriculture and Irrigation of Peru.

### Study area

**Fig 1:**
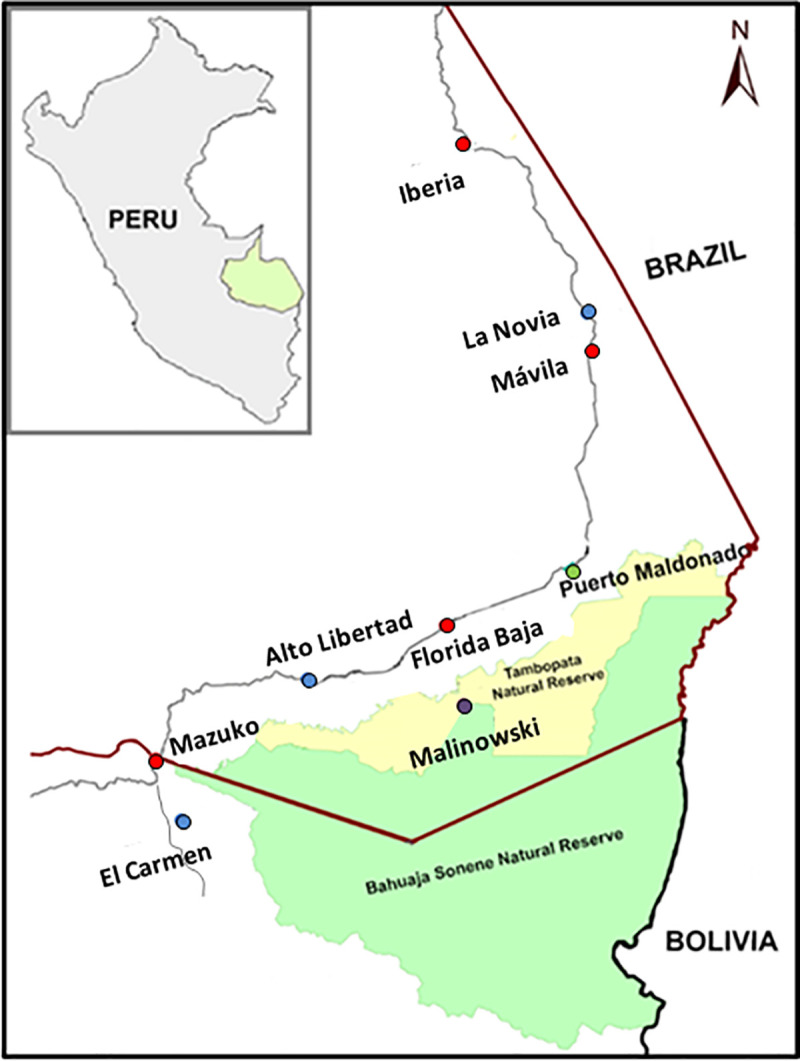
Map of the study area. Figure illustrates the Peru, Brazil and Bolivia border, highlighting Madre de Dios state (Peru) crossed by the interoceanic highway. Study sites are coloured depending on the degree of human impact in red (high impact), blue (low impact) and purple (undisturbed) circles, while the state capital (Puerto Maldonado) is in green. The map was created using open data obtained from OpenStreetMap (CC BY-SA 2.0) URL: https://www.openstreetmap.org/.

The study was conducted in the communities of Iberia, La Novia, Mavila, Alto Libertad, Florida Baja, Mazuko (Madre de Dios, Peru), and El Carmen (Puno, Peru). All communities are located along the Interoceanic Highway route. An additional site (Malinowski), which is located within the limits of Tambopata Natural Reserve and Bahuaja-Sonene National Park (Madre de Dios) was selected as an undisturbed habitat control site (**Fig 1**).

These sites have a humid sub-tropical climate with annual temperatures between 22°C to 34°C and annual precipitation of more than 3,000 mm. The presence of *Leishmania* has been extensively documented in Madre de Dios with reports of *L*. *(V*.*) braziliensis*, *L*. *(V*.*) lainsoni*, *L*. *(V*.*) guyanensis* and *L*. *(V*.*) shawi* from collected human samples [4,10].

### Sand fly collection and taxonomy

**Fig 2:**
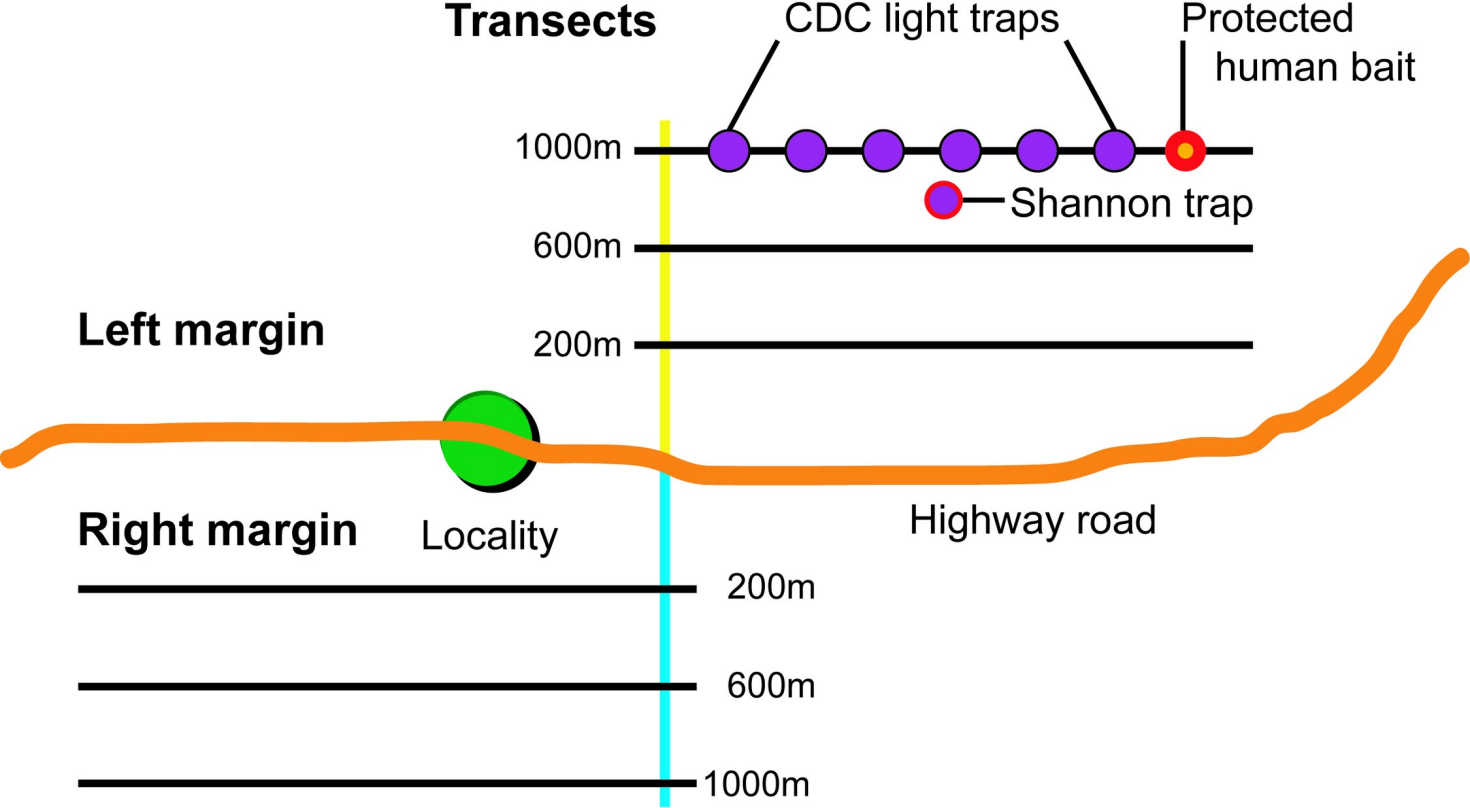
Sand fly trapping approach used in this study. At each study site, collections were performed along three transects (200 m, 600 m and 1,000 m) at the left and right margins of the Interoceanic highway. Six CDC light traps where placed on each transect every 50 m, a Shannon trap was placed at a midpoint between the 600 m and 1,000 m transects and a protected human bait at one end of the transect.

Fieldwork took place between October 2009 and October 2010. In each study site, three 350 m transects were set-up at 200 m, 600 m and 1,000 m from the left and right margins of the highway. All transects were located in settings nearby urban and peri urban areas. Human activities were observed more frequently in the 200 m transects were the secondary forest is predominant; transects at 600 m present intermediate, primary and secondary forest whereas transects at 1,000 m were characterized by the presence of primary forest, wild animals and limited human activity. Sand flies were collected at each site for five consecutive nights using six Mini CDC light traps Model 512 (John W. Hock) placed every 50 m along each transect (**Fig 2**). Protected human bait was performed at one end of each transect and a Shannon trap was placed at the midpoint between the 600 m and 1,000 m transects.

Collected sand flies were stored in 70% ethanol and transported to the NAMRU-6 Entomology Department laboratory in Lima where they were identified based on keys developed by Young and Duncan [11] and Galati [12]. Non-engorged females were pooled in groups of 2–10 specimens or stored individually depending on species, study site, collection date and trap type.

Female specimens were processed using a protocol that allows molecular analysis. Briefly, the head and the two last abdominal segments were separated and placed in lactophenol (Acid Lactic:Phenol 3:2) for two hours at room temperature. These body parts contain key taxonomic structures (cibarium, palpal segments, flagellomeres and spermathecae) which are used for species identification. The remaining parts of the sand fly were preserved in ethanol 70% at -20°C for molecular testing. Male specimens were prepared for mounting and identification with slight modifications from those described by Young & Duncan [11]. Male specimens were placed in 20% KOH for 12 to 24 hours at room temperature. Then, specimens were clarified with lactophenol for two hours at room temperature. Male and female specimens were mounted permanently on Euparal as previously described [13].

### Data analysis

Shannon-Wiener (H) diversity indexes were calculated for each study site including both male and female specimens collected by all trapping methods using the equation $H=-\sum_{i=1}^{n}p\;\ln\;p$ where “p” represents the proportion in which each species “n” was collected (∑p = 1). Statistical significant differences among Shannon-Wiener (H) diversity indexes were assessed by the Hutcheson *t-*test adjusting the false discovery rate using the Benjamini Hochberg method [14,15].

The Index of Species Abundance (ISA)[16] was calculated using the formula *ISA* = (a + Rj)/*k*. This method implies establishing a rank for species abundance per site and then calculating “a” as the number of zero observations for each species in all sites multiplied by “c” which is the single largest rank in all the data set plus 1. The Rj value corresponds to the sum of ranks for a given species in all the sites whereas “k” corresponds to the number of sites. The resulting ISA values were converted into the Standardized Index of Species Abundance (SISA) using the formula *SISA* = (*c* − *ISA*)/(*c* − 1).

Transect based analysis was performed only on data obtained from collections performed at the left side of the highway because not all transects were sampled on the right margin. Comparisons of Shannon Diversity indexes between transects of the same location were conducted using the Hutcheson T-test. Mavila was excluded from the analysis since collections were only performed at the 1,000 m transect.

### DNA extraction and polymerase chain reaction

Pools of less abundant species were not analyzed. In addition, only 50 specimens were analyzed out of pools with more than 50 specimens in a given species, site and transect.

DNA was isolated using the Gentra Puregene kit (QIAGEN, Germantown, US) following the standard manufacturer’s protocol for isolation of genomic DNA.

Detection of *Lutzomyia* DNA was done by targeting the 12S ribosomal DNA using primers T1B 5′-AAA CTA GGA TTA GAT ACC CT-3′ and T2A 5′-AAT GAG AGC GAC GGG CGA TGT-3′ as previously described [17]. Reactions were prepared in 25 μL that contained 1X *Taq* polymerase buffer (Invitrogen, Carlsbad, US), 1.5 mM MgCl_2_, 125 μM dNTPs (Invitrogen, Carlsbad, US), 0.5 μM of each primer, 1 unit of *Taq* DNA polymerase (Invitrogen, Carlsbad, US), and 5 μL of DNA sample. The PCR was run under the following cycling conditions: initial denaturation at 94°C for 5 minutes followed by 35 cycles of denaturation 94°C for 20 sec, annealing at 56°C for 30 sec, and extension at 72°C for 25 sec; and a final extension step at 72°C for 5 min. The reaction generates a product of approximately 400 base pairs (bp).

Detection of *Leishmania* was carried out by amplification of a conserved region of *Leishmania* (*Viannia*) kinetoplast DNA minicircle using the primers MP1-L 5′-TAC TCC CCG ACA TGC CTC TG-3′ and MP3-H 5′-GAA CGG GGT TTC TGT ATG C-3′ as previously described [18]. The final volume of each reaction was 20 μL which contained 1X *Taq* polymerase buffer (Invitrogen, Carlsbad, US), 1.5 mM MgCl_2_, 125 μM dNTPs (Invitrogen, Carlsbad, US), 0.5 μM of each primer, 1 unit of *Taq* DNA polymerase (Invitrogen, Carlsbad, US) and 5 μL of DNA template.

The PCR conditions consisted of an initial denaturation for 5 minutes at 94°C; followed by 35 cycles for 45 seconds at 94°C, 45 seconds at 58°C, and 1 minute at 72°C; and a final extension step for 5 minutes at 72°C. This reaction generated a 70 bp product specific for the *Viannia* subgenus.

Minimum infection rates were calculated for all species with positive *Leishmania* PCR results (number of positive pools/total number of specimens tested x 1,000). This estimate is based on the assumption that positive pools contains one infected sand fly.

## Results

### Sand fly species diversity

During the study period, we collected 9,045 sand flies belonging to the genera *Lutzomyia* (9,022 specimens, 99.7%) and *Brumptomyia* (23 specimens, 0.3%). The subgenera *Psychodopygus* (55.2%), *Nyssomyia* (26.9%) and *Trichophoromyia* (13.9%) were the most abundant. Of all collected sand flies, 4,262 (47.12%) specimens were collected with Miniature CDC light traps, 4,757 (52.59%) with Shannon traps, and 26 (0.29%) with protected human bait. The undisturbed habitat (Malinowski) accounted for 37.6% of all sand fly specimens whereas Iberia accounted for the lowest number of specimens (2.1%).

Species identification using taxonomical keys developed by Young & Duncan (1994) and Galati (2003) resulted in 54 *Lutzomyia* species and one *Brumptomyia* species (**S1 Table)**. Among the *Lutzomyia* species, we report for the first time the presence of *Lutzomyia gantieri* in Peru, collected in the Mazuko site (**S1 Fig**).

Some subgenus *Trichophoromyia* specimens were not identified at the species level due to the high similarity of the female morphology within the subgenus, the lack of male specimens or the detection of multiple circulating species based on male morphology (for instance *Lu*. *auraensis*, *Lu*. *clitella*, *Lu*. *nemorosa*, *Lu*. *sinuosa* and *Lu*. *ubiquitalis*).

The most abundant species in the study were *Lutzomyia davisi* (SISA = 0.948), *Lu*. *carrerai carrerai* (SISA = 0.909), *Lu*. *shawi* (SISA = 0.854), *Lu*. *hirsuta hirsuta* (SISA = 0.774) and *Lu*. *(Trichophoromyia)* spp. (SISA = 0.758) (**S1 Table**).

The most abundant species captured with Shannon traps were *Lutzomyia davisi* (SISA = 0.933), *Lu*. *carrerai carrerai* (SISA = 0.894), *Lu*. *shawi* (SISA = 0.839) and *Lu*. *llanosmartinsi* (SISA = 0.756) whereas *Lu*. *carrerai carrerai* (SISA = 0.866), *Lutzomyia davisi* (SISA = 0.802), *Lu*. *(Trichophoromyia)* spp. (SISA = 0.799) and *Lu*. *shawi* (SISA = 0.732) were the most abundant in CDC light traps.

**Fig 3:**
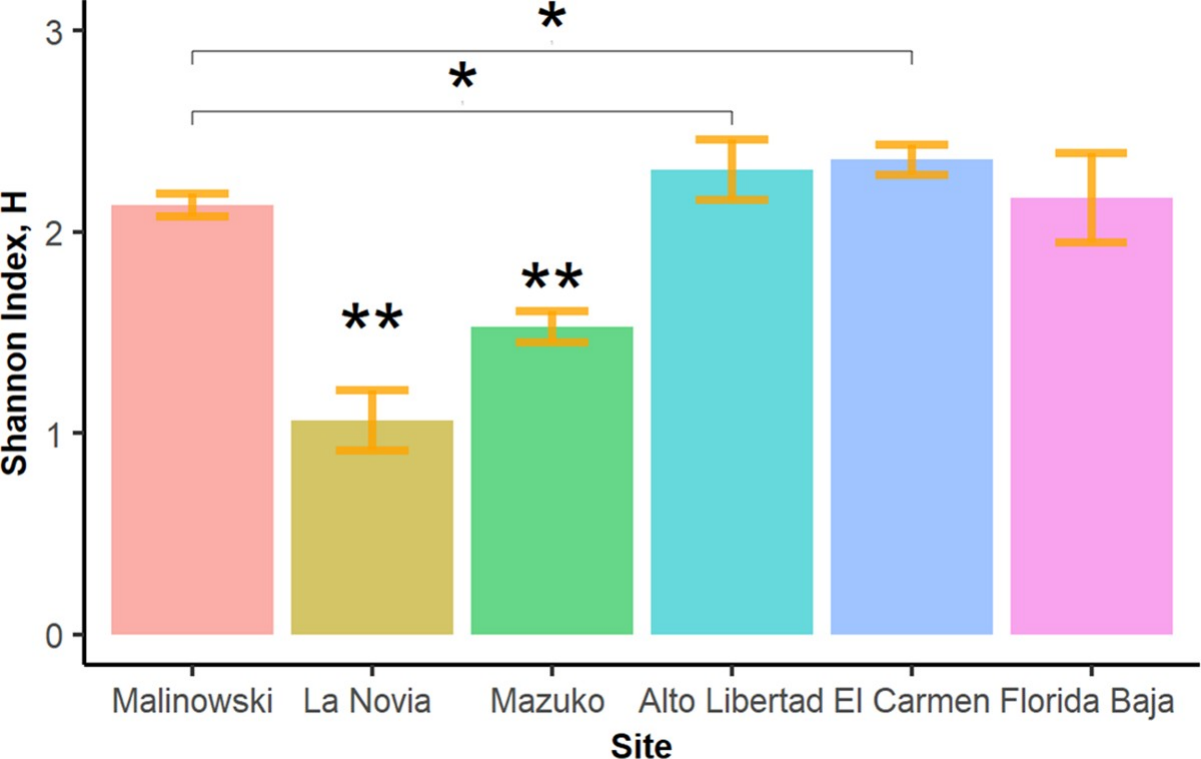
Shannon diversity indexes comparison across study sites. The figure shows that there are significant differences (*<0.05 and **<0.005) on species diversity between all sites versus La Novia and Mazuko, and between Alto Libertad and El Carmen versus Malinowski. Iberia was excluded from this analysis since not all transects were sampled for this site.

La Novia and Mazuko had significantly lower Shannon diversity indexes (H = 1.06 and 1.53, respectively) than all the other sites (FDR adjusted p < 0.05) while Alto Libertad and El Carmen had significantly higher diversity than Malinowski (H = 2.30, 2.36 and 2.13, respectively) (**Fig 3**).

The lower sand fly diversity in the highly disturbed, illegal gold mining sites of La Novia and Mazuko coincided with single species predominance. *Lutzomyia shawi* accounted for 59% of all collected specimens in La Novia, and *Lu*. *carrerai carrerai* for 42% in Mazuko.

Collection methods resulted in significant differences between Shannon diversity indexes estimated with CDC light traps and Shannon traps in El Carmen, Florida Baja and Malinowski (FDR t-test adjusted p < 0.05, **S2 Table)**.

### Transect analysis

**Fig 4:**
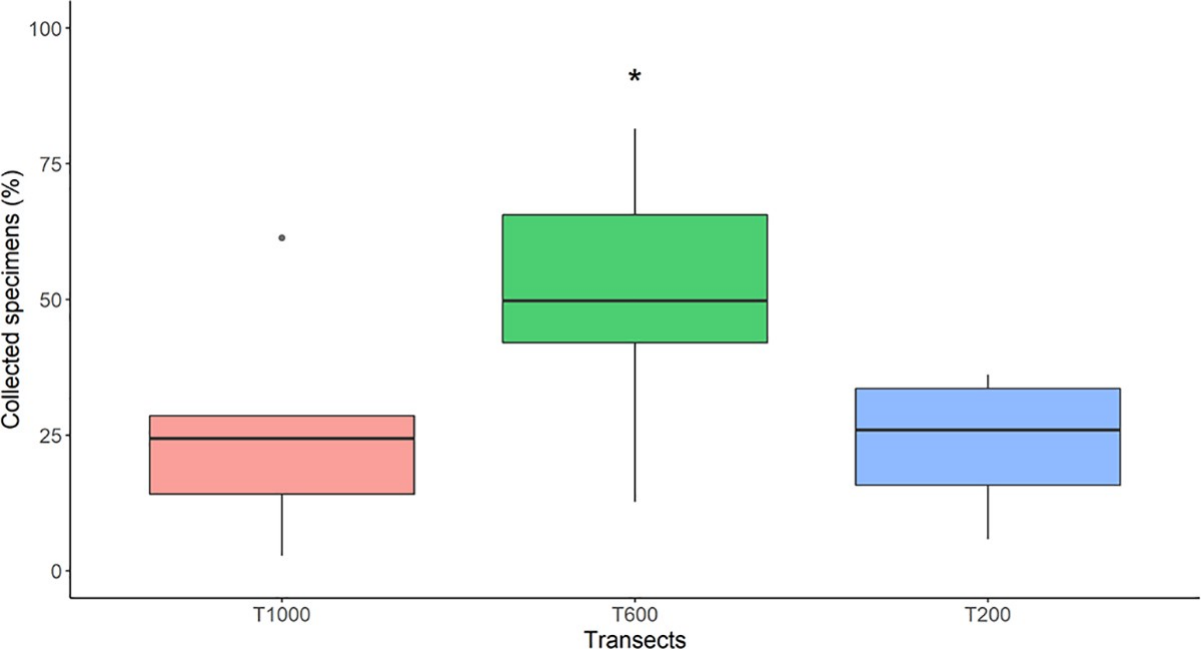
Relative abundance of collected specimens by transect. The figure shows the percentages of collected specimens by transect across sites and denotes a significantly higher sand fly abundance in the 600 m transect versus the other two transects. The dot on the 1,000 m transect denotes an outlier.

Transect based analysis was conducted on collections performed at the left margin of all study sites except for Mavila which was excluded because collections were only performed at the 1,000 m transect, and Iberia where sand fly collections were performed only in the 200 m and 600 m transects. Higher abundance was observed in the 600 m transect compared to each the 200 m and 1000 m transects across all sites (p < 0.05) (**Fig 4**).

*Lutzomyia carrerai carrerai* was the most abundant species in all transects (SISA = 1.000) with higher abundance closer to the road (20.1% at 1,000 m, 37.9% at 600 m and 40.9% at 200 m). The same distribution was found in the second most abundant species, *Lutzomyia davisi* (SISA = 0.910) (6.7% at 1,000 m, 10.5% at 600 m and 15.4% at 200 m), whereas *Lu*. *shawi* (SISA = 0.811) was predominant at 600 m (18.8%), and *Lu*. *yucumensis* (SISA = 0.852) at 1,000 m (11.0%).

**Fig 5:**
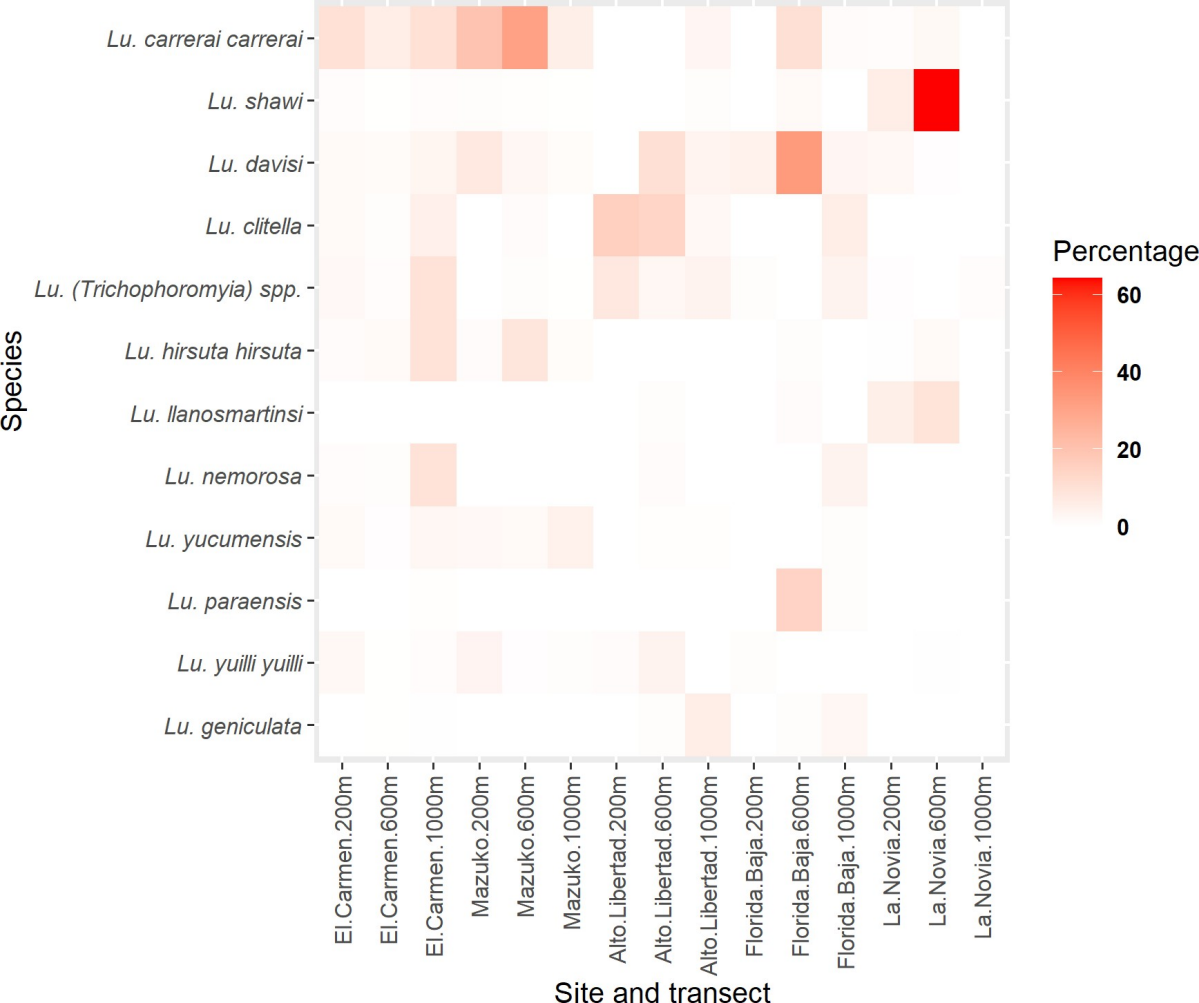
Percentage of collected sand fly species by transect and location. The figure shows the percentages of the most abundant species per transect per each site. Differences were found according to site and transect within and between locations. Statistical significant differences in Shannon diversity indexes between transects of the same location were found in El Carmen (200 m/1,000 m and 600 m/1,000 m), Alto Libertad (200 m/1,000 m and 200 m /600 m) and La Novia (200 m/600 m) (FDR adjusted p<0.05).

The analysis per location and transect showed differences in species abudance in each site (**S3 Table and Fig 5**). *Lutzomyia davisi* (SISA = 0.707), *Lu*. *(Trichophoromyia)* spp. (SISA = 0.679) and *Lu*. *yuilli yuilli* (SISA = 0.671) were the most abundant species at the 200 m transect in El Carmen and Mazuko. *Lutzomyia davisi* (SISA = 0.890), *Lu*. *carrerai carrerai* (SISA = 0.760) and *Lu*. *hirsuta hirsuta* were the most abundant species at the 600 m transect in Mazuko. *Lutzomyia (Trichophoromyia)* spp. (SISA = 0.898), *Lu*. *carrerai carrerai* (SISA = 0.731) and *Lu*. *davisi* (SISA = 0.698) were the most abundant species at the 1,000 m transect in El Carmen and Mazuko.

*Lutzomyia clitella* (SISA = 0.911) and *Lu*.*(Trichophoromyia)* spp. (SISA = 0.889) were the most abundant species at the 200 m transect in Alto Libertad. *Lutzomyia davisi* (SISA = 0.920) was the most abundant at the 600 m transect in Florida Baja whereas *Lu*. *shawi* (SISA = 0.667) was the most abundant at the 600 m transect in La Novia.

### Natural *Leishmania* infections

Non-engorged female sand fly specimens were grouped into 567 pools (56% of the total sand fly females collected) based on species, trap type, transect and site. PCR analysis of the 12S ribosomal DNA confirmed the presence of *Lutzomyia* DNA and ruled out the presence of PCR inhibitors on our samples.

**Fig 6:**
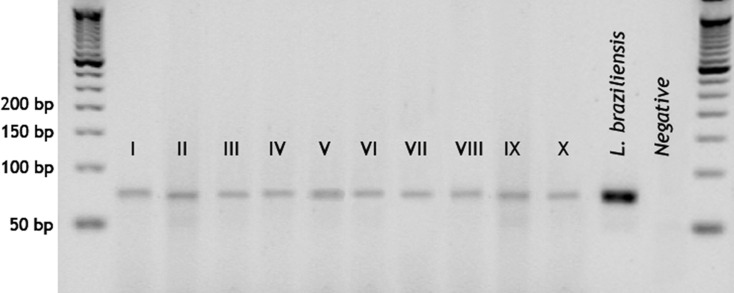
Detection of *Leishmania* DNA on field collected *Lutzomyia* by kinetoplast based PCR. Ten pools were confirmed positive for *Leishmania* by the presence of a characteristic 70 bp band. *Lu*. *(Lutzomyia)* spp. (I), *Lu*. *shawi* (II-IV), *Lutzomyia carrerai carrerai* (V), *Lu*. *yuilli yuilli* (VI), *Lu*. *(Helcocyrtomyia)* spp. (VII and VIII) and *Lu*. *hirsuta hirsuta* (IX and X).

*Leishmania-*specific kinetoplast based PCR detected parasite DNA in 10 pools (1.8%, **Table 1**) from the species *Lu*. *shawi*, *Lu*. *carrerai carrerai*, *Lu*. *yuilli yuilli*, *Lu*. *hirsuta hirsuta* and two subgenera *Lu*. *(Helcocyrtomyia) spp*. and *Lu*. *(Lutzomyia) spp*. (**Fig 6**).

**Table 1:** Positive pools according to *Lutzomyia* species, study site and transect. The table shows that the majority of positive pools were collected in El Carmen, at the 1,000 m transect. The SISA index for each species per locality is also shown and the trap types as CDC Light traps (CDC) or Shannon trap (SH). The last column shows reports of *Leishmania* species naturally infecting these sand fly species [19–23].

Species	Locality	Transect	SISA	Trap type	Species report
***Lu*. *(Lutzomyia)* spp.**	Mazuko	1000	0.225	SH	NA
***Lu*. *shawi***	Florida Baja	1000	0.253	CDC	*L*. *(V*.*) braziliensis* [19]
	El Carmen	200	0.639	SH	
		1000		SH	
***Lu*. *carrerai carrerai***	El Carmen	1000	1.000	SH	*L*. *(V*.*) naiffi* [20]
***Lu*. *yuilli yuilli***	El Carmen	1000	0.721	SH	*L*. *(V*.*) panamensis* [21]; *Leishmania sp*. *siamensis* [20]
***Lu*. *(Helcocyrtomyia)* spp.**	El Carmen	1000	0.238	SH	*Leishmania (V*.*)* spp. [22]
				SH	
***Lu*. *hirsuta hirsuta***	El Carmen	1000	0.891	SH	*Leishmania (V*.*)* spp. [20,23]
				CDC	

El Carmen accounted for eight out of ten positive pools (7 at 1000m, and 1 at 200m transects) whereas the other two positive pools were from Mazuko and Florida Baja (**Table 1**). Shannon traps accounted for most of the positive pools (eight out of ten positive pools) whereas CDC accounted for the remaining two positive pools.

Pools from sand flies collected from 1,000 m transects have a significantly higher *Leishmania* positivity rate than those from 600 m or 200 m transects (9/169 = 5.3% vs 0/79 = 0% and 1/127 = 0.8%, p < 0.05).

The estimated minimum infection rates for *Lu*. *shawi*, *Lu*. *carrerai carrerai*, *Lu*. *yuilli yuilli* and *Lu*. *hirsuta hirsuta* were 0.25%, 0.09%, 0.65% and 0.87% respectively.

## Discussion

The South Eastern Peruvian Amazon is an endemic foci of TL [7,24] that is experiencing dramatic land use changes due to different activities such as illegal mining, logging, agriculture and others that are related to the Transoceanic Hhighway that connects Peru, Brazil and Bolivia.

Given the locations of our study sites along the highway, our results were compared to those obtained in the other side of the border [25,26]. In our study, we found a high density of *Lutzomyia* species of the subgenera *Psychodopygus* (55.4%), *Nyssomyia* (26.0%) and *Trichophoromyia* (13.9%) on the Peruvian side of the border; whereas Teles *et al*. [25] found a higher density of the subgenera *Trichophoromyia* (39.51%), *Psychodopygus* (27.34%), *Pressatia* (9.89%) and *Nyssomyia* (8.58%) in the municipality of Assis, located in Acre (Brazil). A similar study conducted in Rio Branco, Brazil [26] reported a lower number of sand flies and diversity (23 sand fly species) with the subgenera *Trichophoromyia* (53.3%) and *Nyssomyia* (23.5%) as the most prevalent [26].

These comparisons indicate that sand fly distribution and species composition is substantially different between Peruvian and Brazilian border regions. It is possible that environmental and anthropogenic variables including increased urbanization or lower precipitation rates in the Brazilian sites are responsible for these differences [27,28]. Additionally, differences in the study designs, time and year of collection could also explain the differences found.

Our collections showed not only high heterogeneity of sand fly abundance and diversity between regions but also within the same region as shown by the significant differences found at the transect level. These micro-environments with specific sand fly species can be susceptible to anthropogenic change. However, it is important to consider that our study was limited to a single collection at each site and therefore could not assess changes in sand fly diversity and abundance through time.

It has been suggested that residential development in previously forested regions may prompt the spread of TL in these areas [27]. These environmental modifications probably result in shifts in the sand fly population, vector replacement or the introduction of other sand fly species that could in turn transmit the disease [8]. Moreover, the presence of domestic and wild animals in the peridomicile may attract large number of sand flies, thus increasing the risk of transmission [29].

The highest impact of anthropogenic activities was observed in Mazuko and La Novia, where the lowest sand fly species diversity was found. Reduction of species diversity on these sites seemed to happen together the rise of single species predominance (>40% for each *Lu*. *carrerai carrerai* and *Lu*. *shawi*, respectively*)*. Such high abundance suggests that these two sand fly species, which were found to be infected with *Leishmania*, may have adapted well to these areas of transitional ecology.

It has been reported that multiple *Lutzomyia* species are involved in leishmaniasis transmission in the Peruvian-Brazilian Amazon Basin, forming hotspots of multiple infected sand fly species cohabitating in the same region [7,26]. Our study clearly reflects that pattern, with 80% of all positive pools found in the same transect (1,000m) of a single site (El Carmen), and across three distinct *Lutzomyia* species (*Lu*. *yuilli yuilli*, *Lu*. *(Helcocyrtomyia) spp*. and *Lu*. *hirsuta hirsuta*).

We were unable to identify the infecting *Leishmania* species on the positive pools but all the *Lutzomya* species found positive in our study have previous reports of natural *Leishmania* infection.

Previous studies have shown that species of the *Helcocyrtomyia* subgenus such as *Lu*. *peruensis*, *Lu*. *ayacuchensis* and *Lu*. *tejadai* are highly anthropophilic and have major a role in the transmission of *Leishmania (Viannia) spp*. in Peru [22].

*Lutzomyia yuilli yuilli*, *Lu*. *carrerai carrerai*, *Lu*. *shawi* and *Lu*. *hirsuta hirsuta* are widely distributed in the Amazon basin including the border regions of Peru and Brazil. These species are highly anthrophilic and have previously been found infected with *Leishmania (V*.*) spp*. [21,23,26,30,31].

In summary, we found that the sand fly fauna in the Peruvian-Brazilian Amazon Basin is differentially distributed between and within study sites and transects with substantial heterogeneity. Local hot spots of TL transmission appear to occur due to the presence of different species of infected sand flies cohabitating in the same area. Therefore, prevention and control strategies should consider focusing in such areas first to reduce the risk of leishmaniasis transmission. Finally, this study may serve as a basis to better assess the effects that human activities and land use change have in the sand fly fauna and leishmaniasis in the Peruvian southeastern Amazon.

## Supporting information

S1 Fig Lateral view of characteristic paramere and coxite of *Lu*. *Gantieri*. (TIFF)

S1 Table Total sand fly collected and identified along the Interoceanic Highway between 2009–2010. (XLSX)

S2 Table Diversity indexes by locality and trap. The table shows the diversity indexes for each site according to the collection method (Shannon or CDC Light traps). Total indicates the number of female sand flies collected, H indicates the Shannon Diversity Index and S2H its variance.
(XLSX)

S3 Table Species abundance per location and transect. The table shows the number of collected specimens per species at each transect in each study site. Differences were found between sites and within transects at the same site.
(XLSX)
