# Correction: Diversity, distribution and natural *Leishmania* infection of sand flies from communities along the Interoceanic Highway in the Southeastern Peruvian Amazon

**DOI:** 10.1371/journal.pntd.0011077

**Published:** 2023-01-23

**Authors:** Hugo O. Valdivia, Victor O. Zorrilla, Liz. J. Espada, Jocelyn G. Perez, Hugo R. Razuri, Hubert Vera, Roberto Fernandez, Carlos Tong, Bruno M. Ghersi, Gissella M. Vasquez, Roxanne G. Burrus, Andres G. Lescano, Joel M. Montgomery

There is an error in Fig 1. The correct Fig 1 is provided here.

**Fig 1:**
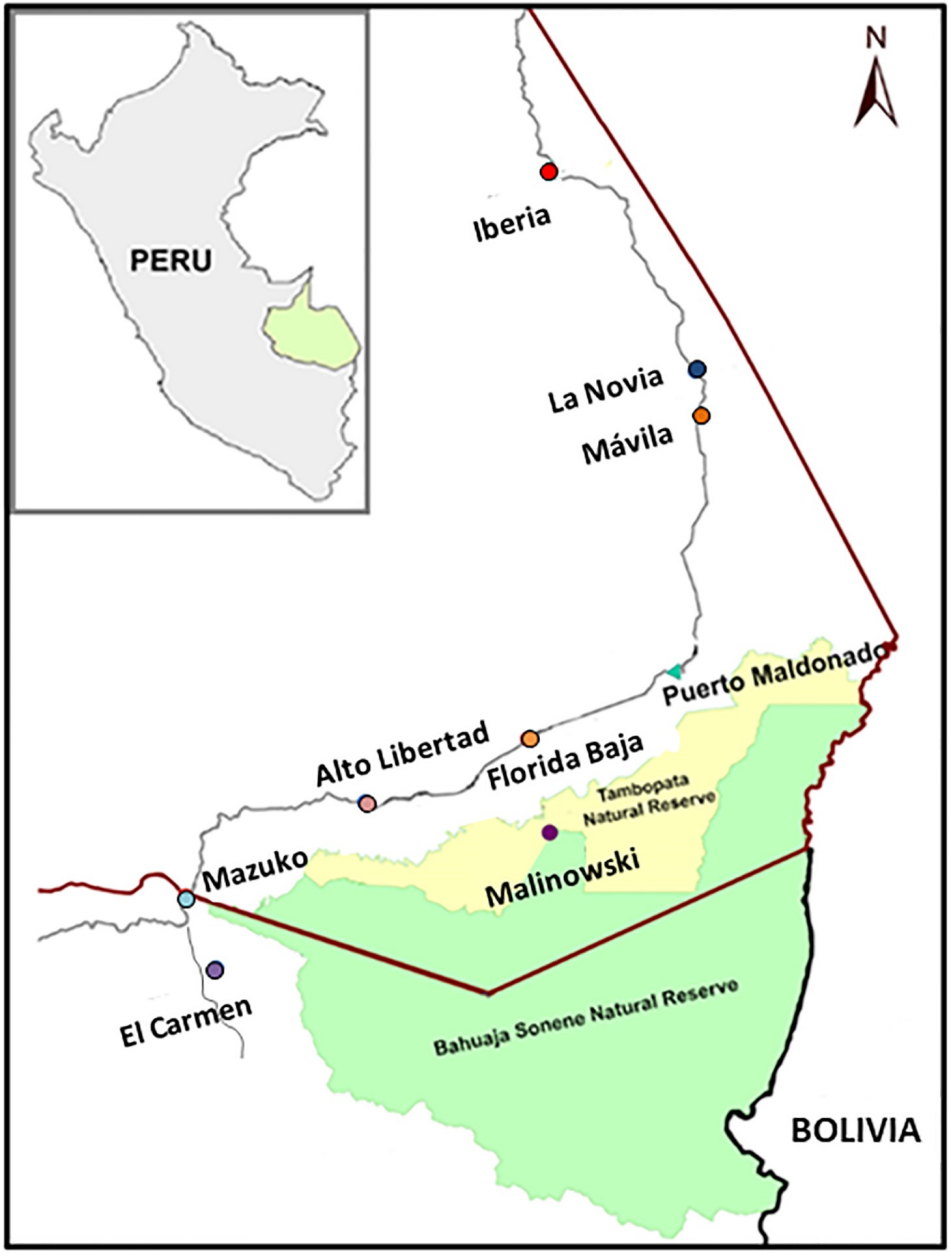
Map of the study area. Figure illustrates the Peru and Bolivia border, highlighting Madre de Dios state (Peru) crossed by the interoceanic highway. Study sites are coloured depending on the degree of human impact in red (high impact), blue (low impact) and purple (undisturbed) circles, while the state capital (Puerto Maldonado) is in green. The map was created using open data obtained from OpenStreetMap URL: https://www.openstreetmap.org/.
