# Supplementary figures and images for: Diversity, distribution and natural Leishmania infection of sand flies from communities along the Interoceanic Highway in the Southeastern Peruvian Amazon
Source: PLoS Negl Trop Dis. 2021 Feb 10;15(2):e0009000. doi: 10.1371/journal.pntd.0009000 (PMC7875382; doi:10.1371/journal.pntd.0009000)

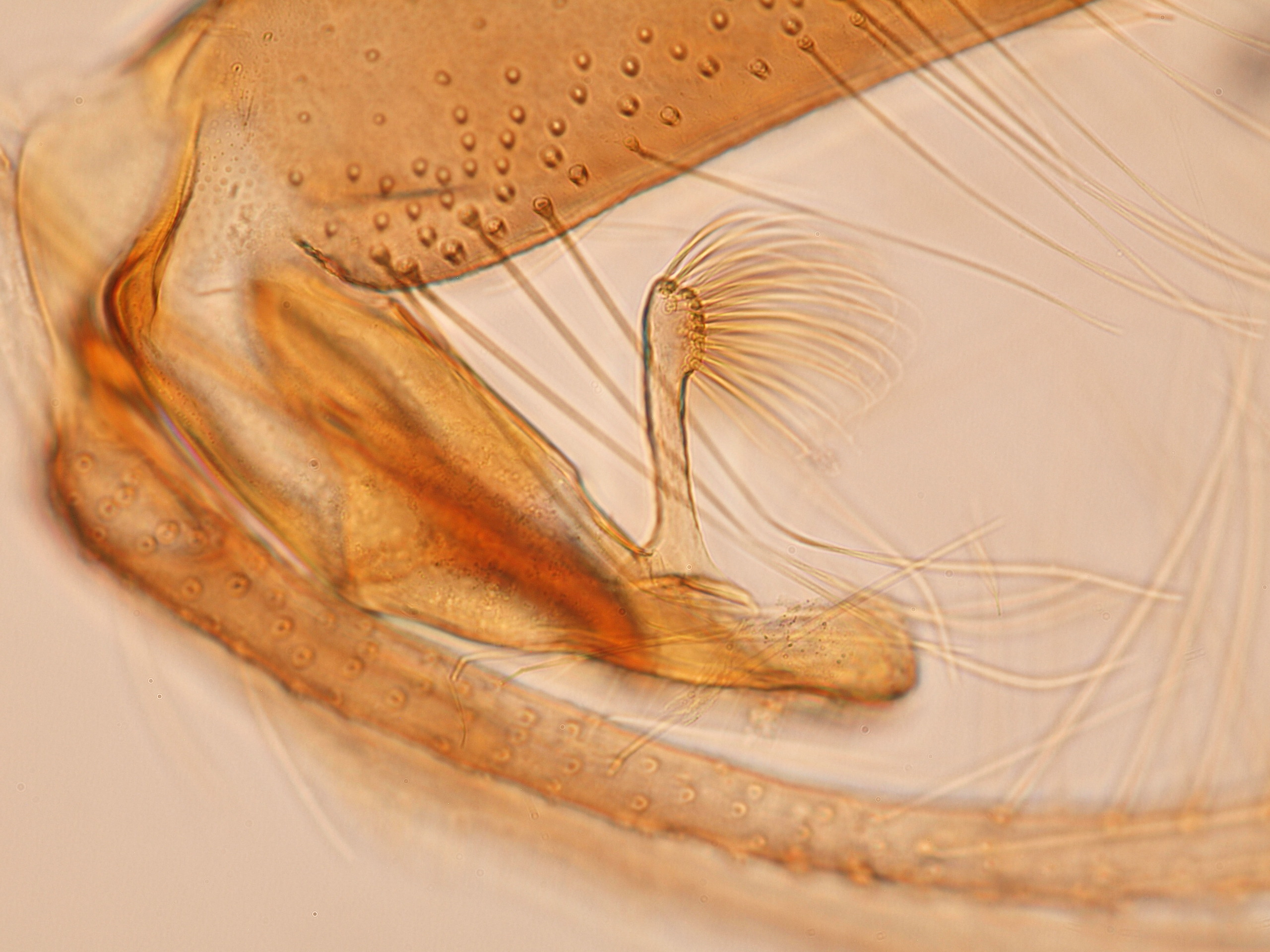

Supplement: S1 Fig — (TIFF) [file pntd.0009000.s001.tiff]
